# The genome of *Austrofundulus limnaeus* offers insights into extreme vertebrate stress tolerance and embryonic development

**DOI:** 10.1186/s12864-018-4539-7

**Published:** 2018-02-20

**Authors:** Josiah T. Wagner, Param Priya Singh, Amie L. Romney, Claire L. Riggs, Patrick Minx, Steven C. Woll, Jake Roush, Wesley C. Warren, Anne Brunet, Jason E. Podrabsky

**Affiliations:** 1 Department of Biology, Center for Life in Extreme Environments, Portland State University, Portland, Oregon USA; 2 Department of Genetics, Stanford University, Stanford, California USA; 3 McDonnell Genome Institute at Washington University, St Louis, Missouri USA; 4 Glenn Center for the Biology of Aging, Stanford, California USA; 5 Knight Cancer Early Detection Advanced Research Center, Oregon Health and Science University, Portland, Oregon USA

**Keywords:** Annual killifish, Stress tolerance, Transcriptome, Gene expression, Fish genome, Positive selection, *Austrofundulus limnaeus*, Development

## Abstract

**Background:**

The annual killifish *Austrofundulus limnaeus* inhabits ephemeral ponds in northern Venezuela, South America, and is an emerging extremophile model for vertebrate diapause, stress tolerance, and evolution. Embryos of *A. limnaeus* regularly experience extended periods of desiccation and anoxia as a part of their natural history and have unique metabolic and developmental adaptations. Currently, there are limited genomic resources available for gene expression and evolutionary studies that can take advantage of *A. limnaeus* as a unique model system.

**Results:**

We describe the first draft genome sequence of *A. limnaeus*. The genome was assembled de novo using a merged assembly strategy and was annotated using the NCBI Eukaryotic Annotation Pipeline. We show that the assembled genome has a high degree of completeness in genic regions that is on par with several other teleost genomes. Using RNA-seq and phylogenetic-based approaches, we identify several candidate genes that may be important for embryonic stress tolerance and post-diapause development in *A. limnaeus*. Several of these genes include heat shock proteins that have unique expression patterns in *A. limnaeus* embryos and at least one of these may be under positive selection.

**Conclusion:**

The *A. limnaeus* genome is the first South American annual killifish genome made publicly available. This genome will be a valuable resource for comparative genomics to determine the genetic and evolutionary mechanisms that support the unique biology of annual killifishes. In a broader context, this genome will be a valuable tool for exploring genome-environment interactions and their impacts on vertebrate physiology and evolution.

**Electronic supplementary material:**

The online version of this article (10.1186/s12864-018-4539-7) contains supplementary material, which is available to authorized users.

## Background

All vertebrates require oxygen and water to complete their life cycles. However, environments are not always forgiving when it comes to constantly providing these basic needs for life. Many metazoans that thrive in highly stressful or variable environments cope by arresting development and entering into states of metabolic depression [1–8]. Examples of organisms able to cope with high environmental stress conditions are typically prokaryotes and invertebrates; extremophile vertebrates are relatively rare. The annual killifish *Austrofundulus limnaeus* (Schultz 1949) is one of the best-described extremophile vertebrates and its embryos have been shown to tolerate low to no oxygen, high and low salinity, ultraviolet-C radiation exposure, and desiccation [9–12]. This stress-tolerant phenotype is essential for survival in tropical and subtropical ephemeral ponds that experience periods of daily temperature fluctuations, seasonal habitat desiccation, anoxia, and hypoxia [13, 14]. Because adult annual killifishes are unable to endure the dry season, populations persist due to the survival of drought-tolerant embryos that can depress metabolism in a state of suspended animation, known as diapause [7, 9]. Diapause may occur at three distinct stages of development in annual killifishes, termed diapause I, II, and III [15–17]. Diapause I (DI) can occur early in development after completion of epiboly but prior to the formation of the embryonic axis. Contrasting with the typical pattern of convergence and extension of the amoeboid (deep) embryonic blastomeres that is observed in most other teleost embryos during epiboly, deep blastomeres of *A. limnaeus* dissociate and migrate away from each other across the yolk surface during epiboly [18, 19]. Essentially, this process appears to temporally disconnect epiboly from germ layer formation in annual killifishes and these deep cells can remain dispersed across the yolk surface for several days before reaggregating and forming a definitive embryonic axis [16]. Although the biological significance and mechanism of this phenomenon are still unclear, it has been suggested that the spatial arrangement of embryonic cells in DI may allow damaged cells to be “sloughed” and replaced by surrounding pluripotent cells [12, 16]. This phenomenon may be facilitated by unique expression of genes important for gastrulation [20]. Diapause II (DII) occurs in the long-somite embryo approximately midway through development, just prior to the major phases of organogenesis, and appears to be the most stress-resistant diapause stage [15, 17, 21]. Finally, embryos can arrest as a late pre-hatching embryo in diapause III (DIII) rather than immediately hatching [17, 22]. While the majority of *A. limnaeus* embryos will enter into DII, a small proportion of “escape” embryos will bypass DII and instead develop directly to DIII or hatching [17, 23]. Importantly, entrance into diapause is an alternative developmental trajectory that is unique biochemically, physiologically, and morphologically from escape embryos.

The unique biology of *A. limnaeus* and other annual killifishes provides an exceptional opportunity to study questions related to stress physiology, development, and evolution by making comparisons within species and across species [2, 21, 24]. One of the best-studied examples of *A. limnaeus* as an important and unique model organism stems from the extreme anoxia tolerance of its embryos, having the highest known tolerance compared to any other vertebrate after temperature is considered [11]. In contrast, insufficient oxygen quickly leads to disruption of cellular homeostasis and cell death in mammals during similar developmental stages, and especially in adult mammalian tissues [25, 26]. This tolerance in *A. limnaeus* embryos peaks at DII (LD_50_ anoxia = 65 days) and is retained for up to 4 days post-diapause (dpd) [11]. Importantly, 4 dpd embryos are physiologically and developmentally distinct from DII embryos, as they have reentered the cell cycle and are metabolically active, and therefore are a model that can have broader implications for comparative studies to other vertebrates. Although the metabolic and cell cycle mechanisms that may support extreme anoxia tolerance and post-DII (PDII) development have previously been explored [11, 27–29], a comprehensive study of the genetic, evolutionary, and gene expression mechanisms that may support the unique features of *A. limnaeus* and other annual killifishes has yet to be performed. Recent work on the African annual killifish, *Nothobranchius furzeri*, suggests that positive selection on *N. furzeri* genes may have a role in determining the annual killifish phenotype [30, 31]. Because it has long been hypothesized that annualism was evolved independently several times within the killifish families Rivulidae and Nothobranchidae [32–34], determining if the evolutionary mechanisms are shared between different annual killifish lineages will have broad implications for the understanding of complex phenotype evolution.

The development of an *A. limnaeus* genomics resource is essential for understanding the evolutionary background of its unique phenotype, and is a necessary tool for functional research such as RNA-seq based expression studies. In this work we present the first de novo draft genome assembly and annotation of a lab reared *A. limnaeus* individual originating from a population near the town of Quisiro, in the Maracaibo Basin of Venezuela [35]. This population has been bred under laboratory conditions since 1995 and is currently maintained at the Center for Life in Extreme Environments Aquatics Facility, Portland State University, Portland, OR. We show for the first time the gene expression profiles that support extreme anoxia tolerance and post-diapause development, as well as potential genes under positive selection in the *A. limnaeus* lineage compared to several teleost species. The resulting genome and annotation is the first publicly available representative of a South American annual killifish and we discuss several genes of interest for future studies in stress tolerance and vertebrate development.

## Results

### Estimation of genome size

**Fig. 1:**
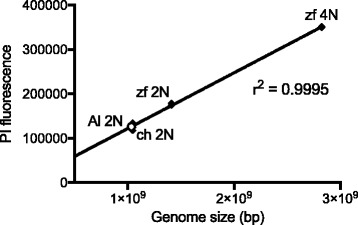
Linear relationship between genome size and propidium iodide (PI) fluorescence. Nuclei were extracted from chicken and zebrafish blood to infer the genome size of the *A. limnaeus* genome. Replicates are plotted as individual data points and are overlapping within the *A. limnaeus* 2 N and zebrafish 4 N points (*n* = 3 for chicken and *A. limnaeus*, *n* = 2 for zebrafish 2 N and 4 N). The *A. limnaeus* data point is an open circle. Zf, zebrafish; ch, chicken; Al, *A. limnaeus*

Flow cytometry quantification of propidium iodide fluorescence intensity yields an estimated genome size for *A. limnaeus* of 1.03 Gbp when *Danio rerio* (zebrafish) and *Gallus gallus* (domestic chicken) free-nuclei were used as standards (Fig. 1). *A. limnaeus* genome size was also estimated by k-mer frequency in Allpaths-LG [36]. This sequence-based method estimated the genome at 974 Mbp, about 5% smaller than the flow cytometry estimate (Table 1).

**Table 1:** Species comparison of total estimated genomic GC and repeat content

Species	Genome size^a^ (gb)	GC%	Repetitive%	References
*A. limnaeus*	0.974–1.03	41.6	46	This work
Zebrafish	1.41	50.6	52.2	[94]
*N. furzeri*	1.3–1.9	44.9	45	[40] [30]
Stickleback	0.461	50.5	6.6	[95]
Cod	0.83	50.0	25.4	[96]
Human	3.1	46.1	66–69	NSDC Assembly GCA_000001405.17, [97, 98]

^a^The estimated genome size ranges of *A. limnaeus* and *N. furzeri* are based on flow cytometry and computational methods. For all others, the genome sizes are based on total assembly length

### Genome assembly and annotation results

To provide a high-quality genome for annotation, we used a merged assembly strategy that utilized mate-pair libraries for scaffolding. Of the initial read pool used as input for Allpaths-LG, the assembler used 65.6% and 28.6% of the fragment and jumping libraries, respectively, for the initial assembly of the *A. limnaeus* genome at a sequencing coverage of 94X. This Allpaths-LG assembly produced 141,049 contigs longer than 1000 bp, and within these contigs 650 Mbp was assembled. JR-assembler assembled 370,267 contigs (> 500 bp) with an N50 of 1.3 kbp and a total assembled length of 433 Mbp. After merging the two assembles with a graph accordance assembly program [37], the *A. limnaeus* draft genome assembly v1.0 has 168,369 contigs, has a contig N50 of 8.1 kb, and is 695 Mbp in total length. Use of mate-pair libraries for scaffolding with SSPACE organized contigs into 29,785 scaffolds, with an N50 of 1.1 Mbp, and increased the total sequence length to 867 Mbp, including gaps (Table 2). Based on the results of our genome size estimates, between 84 and 89% of the *A. limnaeus* genome is represented in the current draft genome assembly. Core Eukaryotic Genes Mapping Approach (CEGMA) results indicate that 68.97% (complete) and 95.96% (partial/complete) of 248 highly conserved eukaryotic genes are present in the assembly (Table 3). Benchmarking Universal Single-Copy Orthologs (BUSCO) analysis using 3023 conserved vertebrate genes indicates that 75% of these are completely present in the assembly, 1.6% are duplicated, 12% are fragmented, and 11% are missing (Table 4). This draft assembly *Austrofundulus limnaeus* 1.0 (GenBank accession GCA_001266775.1) was annotated using the NCBI Eukaryotic Genome Annotation Pipeline. A total of 9.3 billion RNA-seq reads were aligned to the *A. limnaeus* genome at an average alignment frequency of 73%. This automated pipeline was able to annotate 26,157 genes and is publicly available on NCBI as the *Austrofundulus limnaeus* Annotation Release 100. The complete feature results of the NCBI Eukaryotic Genome Annotation Pipeline for *A. limnaeus* are shown in Tables 5 and 6.

**Table 2:** Genome assembly metrics for the *A. limnaeus* genome version 1.0

Metric	Length/number
Total sequence length	866,963,281 bp
Total assembly gap length	171,917,903 bp
Number of scaffolds	29,785
Scaffold N50	1,098,383 bp
Scaffold L50	184 bp
Number of contigs	168,369
Contig N50	8097 bp
Contig L50	24,012 bp

**Table 3:** CEGMA analysis report comparing completeness of different teleost genomes

Species	Database	Assembly	Percent complete	Percent partial or complete
*Austrofundulus limnaeus*	NCBI	GCA_001266775.1	68.97% (171/248)	95.96% (237/248)
*Nothobranchius furzeri* (FLI)	NCBI	GCA_001465895.2	79.44% (197/248)	96.77% (240/248)
*Nothobranchius furzeri* (Stanford)	NCBI	GCA_000878545.1	76.61% (190/248)	96.77% (240/248)
*Fundulus heteroclitus*	NCBI	GCA_000826765.1	79.44% (197/248)	96.77% (240/248)
*Gadus morhua*	Ensembl	gadMor1	63.71% (158/248)	95.97 (238/248)
*Gasterosteus aculeatus*	Ensembl	BROAD S1	86.29% (214/248)	97.58 (242/248)
*Kryptolebias marmoratus* ^*a*^	–	–	83.87% (208/248)	95.16% (236/248)

^a^CEGMA results for *K. marmoratus* are from Kelley et al. [99]

**Table 4:** BUSCO analysis report comparing completeness of different teleost genomes

Species	Database	Assembly	BUSCO database	Complete	Duplicated	Fragmented	Missing	n
*Austrofundulus limnaeus*	NCBI	GCA_001266775.1	vertebrata	75%	1.6%	12%	11%	3023
*Nothobranchius furzeri* (FLI)	NCBI	GCA_001465895.2	vertebrata	76%	1.3%	13%	9.3%	3023
*Nothobranchius furzeri* (Stanford)	NCBI	GCA_000878545.1	vertebrata	73%	1.8%	15%	11%	3023
*Danio rerio*	Ensembl	GRCz10	vertebrata	80%	3.4%	12%	6.1%	3023
*Fundulus heteroclitus*	NCBI	GCA_000826765.1	vertebrata	80%	1.8%	11%	8.3%	3023
*Gadus morhua*	Ensembl	gadMor1	vertebrata	69%	1%	17%	13%	3023
*Gasterosteus aculeatus*	Ensembl	BROAD S1	vertebrata	85%	2.3%	9.70%	4.8%	3023
*Kryptolebias marmoratus* ^a^	–	–	vertebrata	76.5%	1.6%	10.20%	13.3%	3023

^a^*K. marmoratus* BUSCO results were obtained from Kelley et al. [99]

**Table 5:** NCBI Eukaryotic Genome Annotation Pipeline feature report for the *A. limnaeus* genome

Feature	Count	Mean length (bp)	Median length (bp)	Min length (bp)	Max length (bp)
Genes	26,157	18,385	8286	71	638,625
All transcripts	38,958	2868	2288	71	88,923
*mRNA*	35,329	3063	2454	183	88,923
*misc_RNA*	370	2747	2371	115	13,579
*tRNA*	441	74	73	71	87
*lncRNA*	2818	884	676	89	6645
Single-exon transcripts	1638	1516	1291	183	7265
*coding transcripts (NM_/XM_)*	1638	1516	1291	183	7265
CDSs	35,329	2011	1434	96	87,576
Exons	246,940	264	137	2	17,283
*in coding transcripts (NM_/XM_*)	239,150	262	136	2	17,283
*in non-coding transcripts (NR_/XR_*)	10,381	281	140	2	7351
Introns	215,079	2084	353	8	369,543
*in coding transcripts (NM_/XM_*)	210,044	2037	351	8	369,543
*in non-coding transcripts (NR_/XR_*)	7596	3392	463	30	180,124

**Table 6:** Support for gene models using the NCBI Eukaryotic Genome Annotation Pipeline

Feature	Count
Genes and pseudogenes	26,712
*protein-coding*	23,844
*non-coding*	2313
*pseudogenes*	555
*genes with variants*	6136
mRNAs	35,329
*fully-supported*	33,945
*with > 5%* ab initio	552
*partial*	11,187
*with filled gap(s)*	10,926
*known RefSeq (NM_)*	0
*model RefSeq (XM_)*	35,329
Other RNAs	3629
*fully-supported*	3188
*with > 5%* ab initio	0
*partial*	36
*with filled gap(s)*	36
*known RefSeq (NR_)*	0
*model RefSeq (XR_)*	3188
CDSs	35,368
*fully-supported*	33,945
*with > 5%* ab initio	639
*partial*	9918
*with major correction(s)*	3568
*known RefSeq (NP_)*	0
*model RefSeq (XP_)*	35,329

Using k-mer frequency, Allpaths-LG predicted the *A. limnaeus* genome to have a G + C content of 41.6%, an overall single nucleotide polymorphism rate of 1 in 147 bases, and to be 46% repetitive (Table 1). The total repeat content in the *A. limnaeus* genome is much higher than other teleost species such as *Gasterosteus aculeatus* (stickleback) and *Gadus morhua* (cod), but is very similar to *N. furzeri* and *D. rerio* (Table 1). RepeatModeler was able to detect approximately 30% of the assembled genome as repetitive elements (Table 7).

**Table 7:** Identified repeat classes in *A. limnaeus* using RepeatModeler and compared to zebrafish (*D. rerio*)

	*A. limnaeus*		*D. rerio*	
Repeat Masker %	27.55		–	
Tandem repeat finder %	3.13		–	
Total % identified^a^	30.68		52.2	
Repeat class/superfamily^a^				Type
SINE %	0.69		2.216	type I transposons
LINE %	3.89		2.58	type I transposons
LTR elements %	0.77		3.314	type I transposons
non-LTR elements	–		2.495	type I transposons
DNA elements %	6.36		38.51	type II transposons
Unclassified	15.22		0.05	
Simple repeats %	0.42		6.44	
Low complexity	0.42		10.68	

^a.^Repeat categories are listed as percentages of their respective whole genomes

### Positive selection in *A*. *limnaeus* genes associated with mitochondrial function

**Fig. 2:**
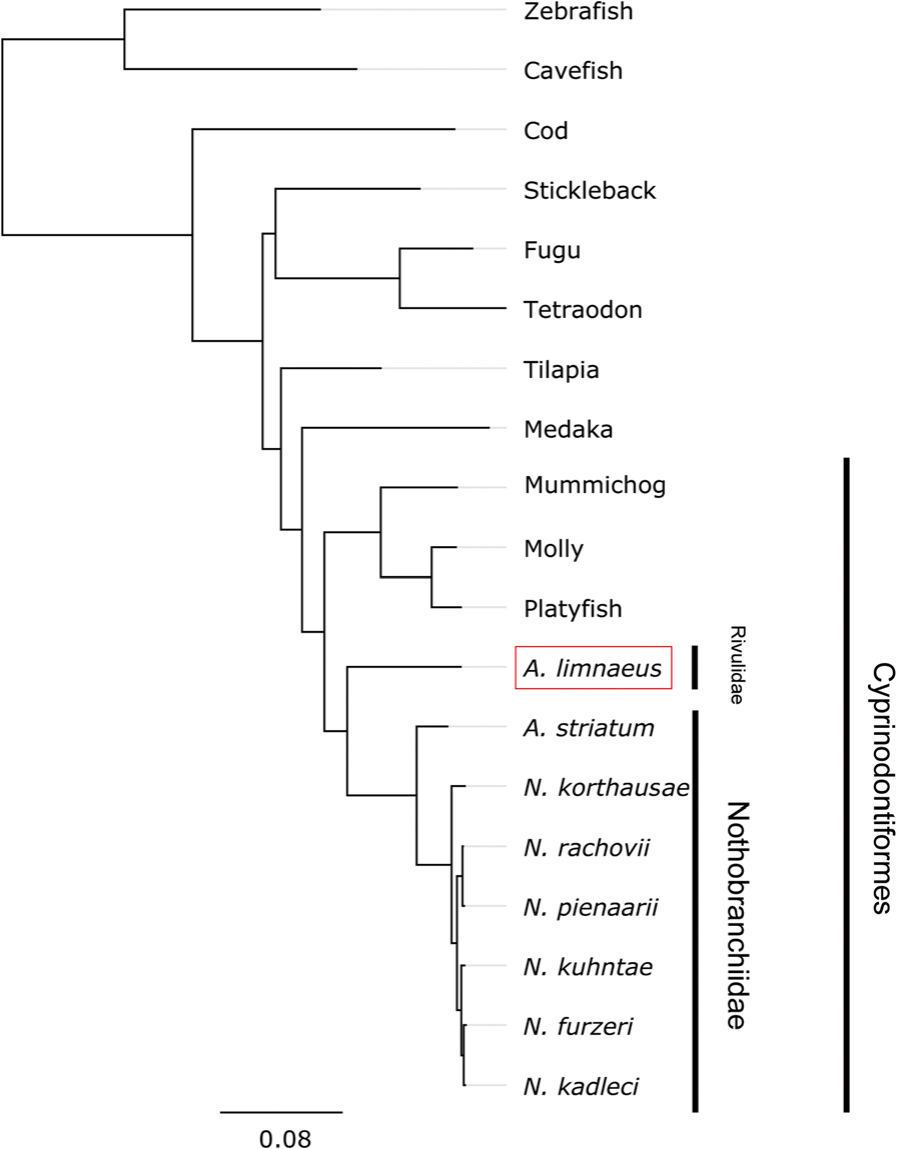
Rooted phylogenetic tree of the species used for PAML analysis. The tree was inferred using PhyML with 100 bootstrap replications. A GBLOCKS-filtered alignment of highly conserved coding genes was used as input (see Materials and Methods). All nodes have 100% bootstrap support. Cavefish = *Astyanax mexicanus*, Cod = *Gadus morhua*, Fugu = *Takifugu rubripes*, Medaka = *Oryzias latipes*, Molly = *Poecilia formosa*, Mummichog = *Fundulus heteroclitus*, Platyfish = *Xiphophorus maculatus*, Stickleback = *Gasterosteus aculeatus*, Tetraodon = *Tetraodon nigroviridis*, Tilapia = *Oreochromis niloticus*, Zebrafish = *Danio rerio*

**Fig. 3:**
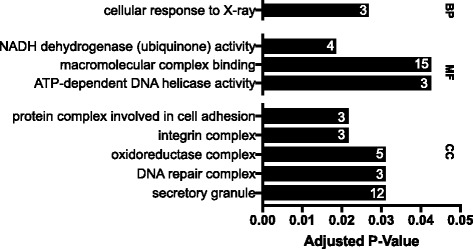
Significantly enriched GO terms for genes under putative positive selection (adjusted *P*-value ≤0.05). The numbers within the bars indicate the total number of *A. limnaeus* genes associated with the GO term that were determined to be under putative positive selection following PAML analysis. BP, biological process; MF, molecular function; CC, cellular component

**Fig. 4:**
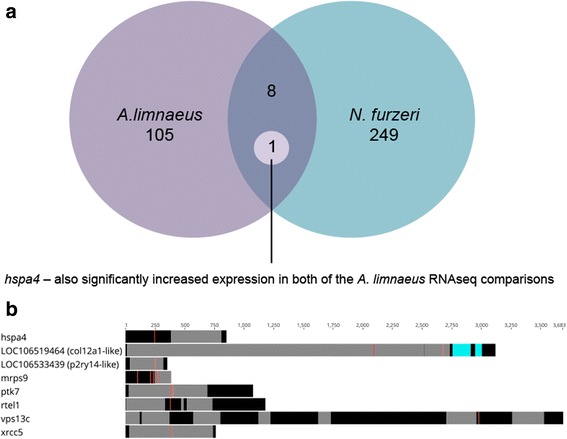
Genes under positive selection in both *N. furzeri* and *A. limnaeus*. **a** Eight orthologs have at least one site under putative positive selection in both *A. limnaeus* and *N. furzeri*. One of these shared orthologs is hspa4, which was also found to be significantly increased in expression during anoxia in DII embryos and in post-DII embryos compared to DII controls. **b** Putative *A. limnaeus* positively selected sites of these eight orthologs occurs both within predicted domains and between them. Numbers on the X-axis refer to amino acid location. Red, selected site; Black, conserved domain predicted by InterProScan 5; Light blue, repeat domain

**Fig. 5:**
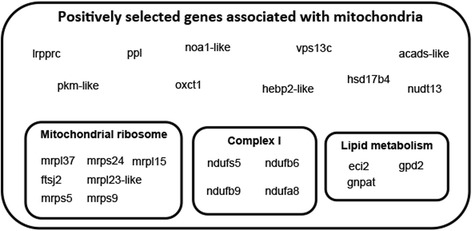
Putative positively selected proteins in *A. limnaeus* that are associated with mitochondrial biogenesis or function. Of the 24 genes associated with mitochondria, seven are associated with the mitochondrial ribosome, four are associated with complex I of the electron transport chain, and three are associated with lipid metabolism related to mitochondria

To identify genes that may support the unique biology of *A. limnaeus*, we utilized a comparative approach to find genes under possible positive selection in the lineage. We collected highly conserved protein coding genes from several teleost species and generated a phylogenetic tree with strong node support (Fig. 2; Additional file 1: Table S1A). *A. limnaeus* is sister to the Family Nothobranchiidae, which includes the African annual killifish genus *Nothobranchius* as well as the non-annual African killifish *A. striatum*. Of the 4152 gene families that passed our filters for PAML analysis, 105 genes were determined to have at least one significantly selected site along the *A. limnaeus* branch (FDR < 0.2, Additional file 1: Table S1B). Several GO terms within the biological process, molecular function, and cellular component categories are significantly enriched among this list of genes (adjusted *P* ≤ 0.05, Fig. 3; Additional file 1: Table S1C). To further interrogate these 105 genes, we compared the list to 249 genes previously reported by Valenzano et al. to be under putative positive selection along the *N. furzeri* branch [30]*.* Of these 249 genes, eight orthologs were also called by PAML as having at least one site under selection in *A. limnaeus* (Fig. 4a,b). Overall, we found that 24 out of 105 (23%) of the genes under putative positive selection in *A. limnaeus* annotated to nuclear genes associated with mitochondrial biogenesis or activity (Fig. 5).

### Differential expression of DII RNAs following anoxia or exit from DII

**Fig. 6:**
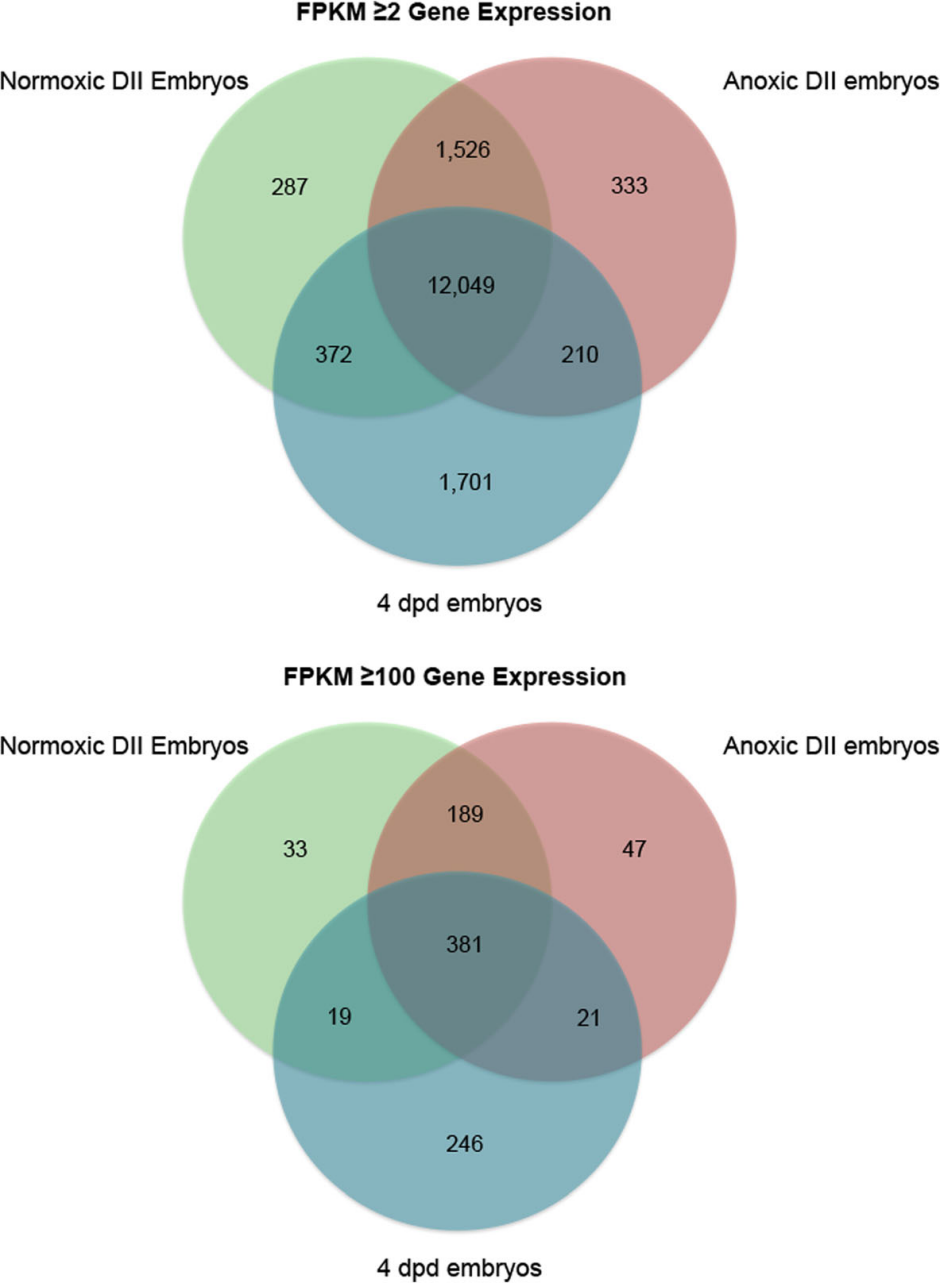
Overlap in the expression of genes between the three groups examined in this work. Venn diagrams are shown at FPKM thresholds of ≥2 (top) and ≥100 (bottom) to compare the normoxic DII, anoxic DII, and 4 dpd embryo RNA-seq data sets used in this work

Among the three embryo groups used in this study (DII normoxia, DII 24 h anoxia, 4 dpd normoxia) we found that 12,049 and 381 genes were shared between the groups at abundance thresholds of Fragments Per Kilobase of transcript per Million mapped reads (FPKM) ≥ 2 and FPKM ≥100, respectively (Fig. 6).

#### Gene expression of DII embryos in anoxia

**Fig. 7:**
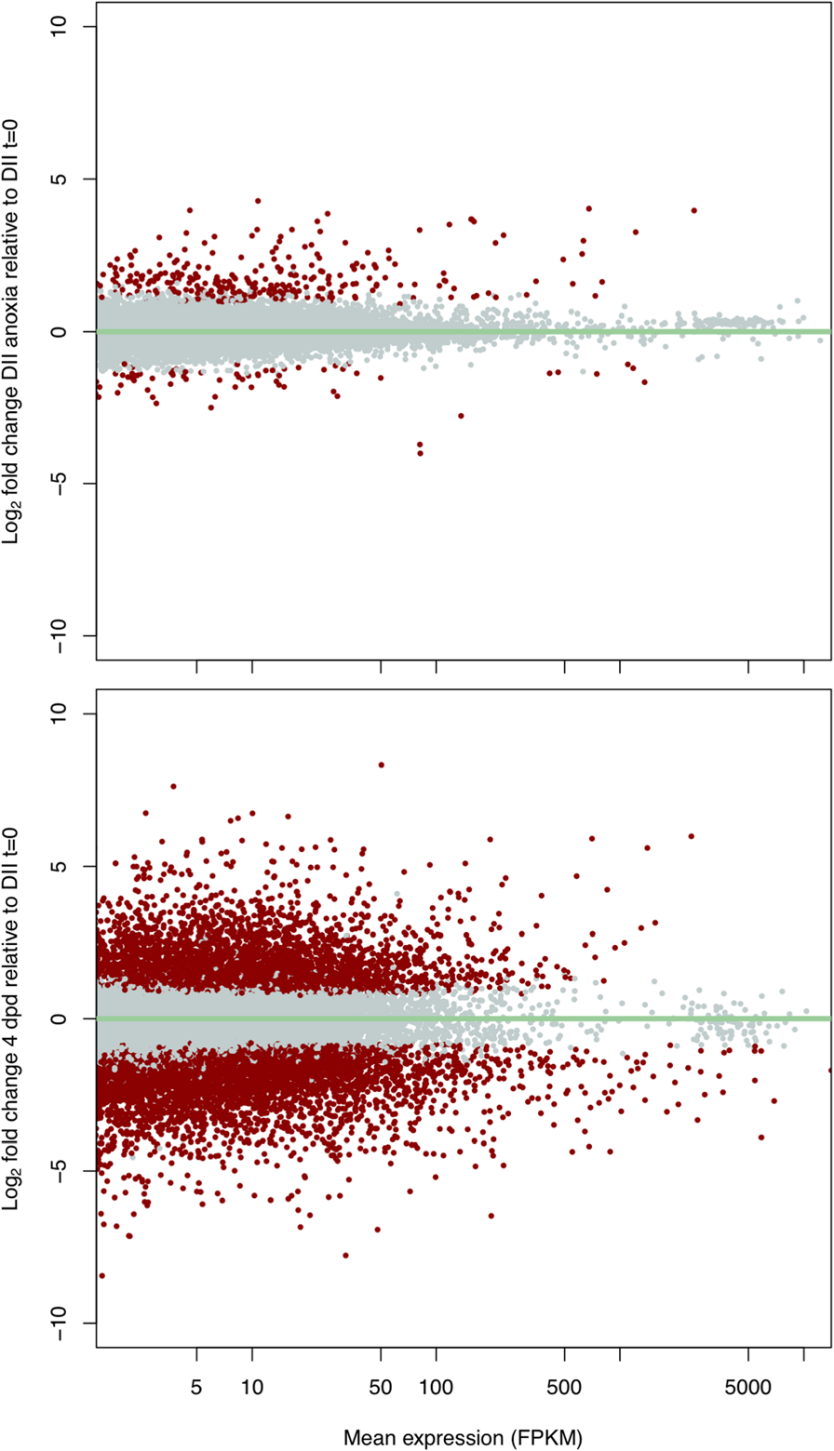
Differential abundance of anoxic DII embryos and 4 dpd embryos relative to normoxic DII embryos. Significant abundance changes in transcripts are seen in anoxic DII embryos (top) and in 4 dpd embryos (bottom) when compared to normoxic DII embryos. Transcripts in grey do not have a significant difference in abundance. Transcripts in red are significant (FDR adjusted *p*-value < 0.05). FPKM, Fragments Per Kilobase of transcript per Million mapped reads

**Fig. 8:**
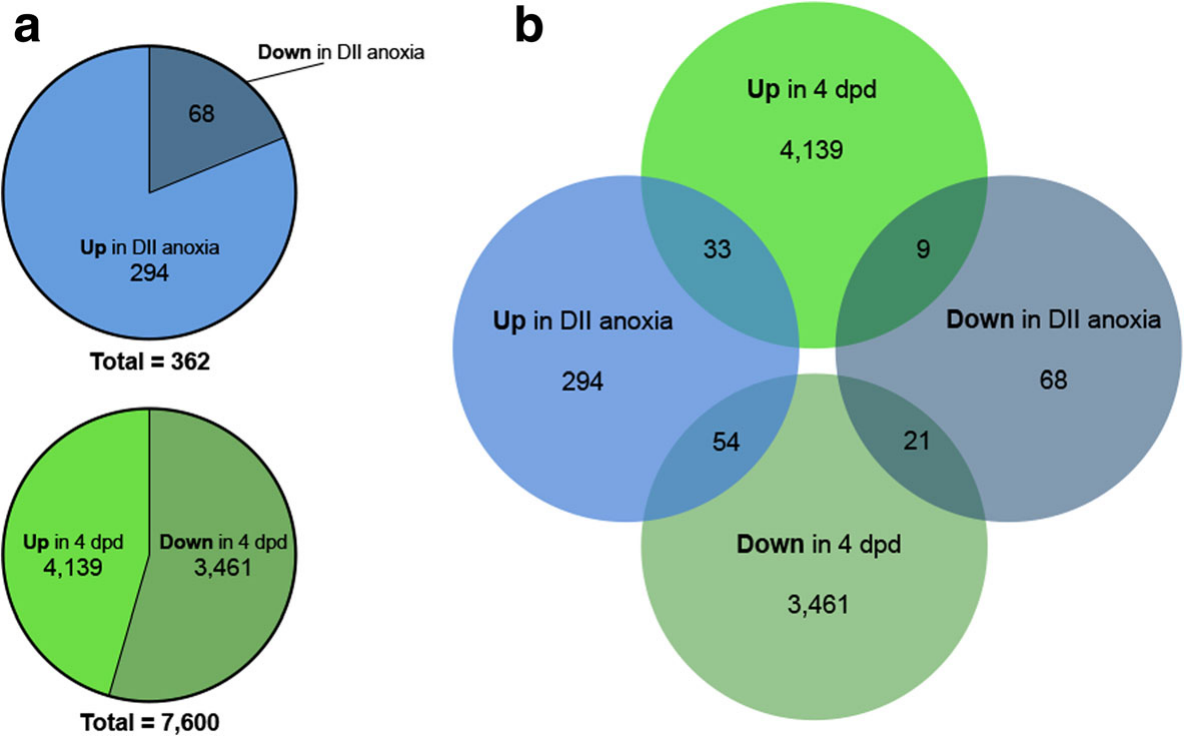
Summary of *A. limnaeus* embryo differential transcript abundance analysis. **a** Transcripts that change significantly in abundance relative to normoxic DII embryos (FDR adjusted *p*-value < 0.05). **b** The number of significantly differentially expressed transcripts that overlap among possible comparisons

**Fig. 9:**
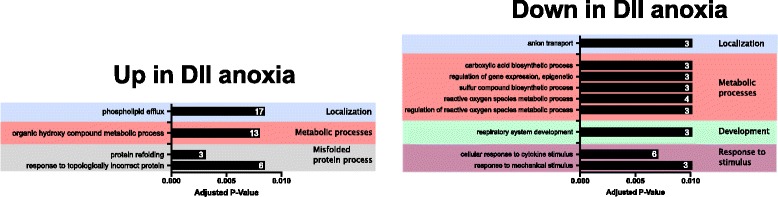
Significantly enriched GO terms based on differential abundance analysis of anoxic and normoxic DII embryos. Only terms with an adjusted P of < 0.01 are shown. Numbers in the bars indicate the total number of transcripts that change in abundance associated with the respective GO term

Following 24 h of anoxia in DII embryos, the vast majority of transcripts do not change in abundance. Of the transcripts expressed that reached our FPKM threshold, 294 transcripts increased in abundance, while 68 transcripts decreased in abundance (false discovery rate, FDR adjusted *P* < 0.05; Fig. 7, Fig. 8A; Additional file 1: Table S1D). Several of the transcripts with the largest increases in FPKM included proteins involved in the heat shock response (Table 8). GO terms for genes that increased in abundance were enriched for localization, metabolism, and the misfolded protein process (adjusted *P* < 0.01, Fig. 9; Additional file 1: Table S1E). Of the transcripts that significantly decreased in abundance, the top four with the largest decreases in FPKM relative to normoxia are all components of complex I, and all ten are transcripts associated with mitochondria (Table 8). Significantly enriched GO terms for genes that decreased in transcript abundance showed enrichment for localization, metabolism, development, and response to stimulus (adjusted *P* < 0.01, Fig. 9; Additional file 1: Table S1F).

**Table 8:** Top 10 genes in anoxic DII embryos with greatest transcript abundance changes relative to normoxia

	Gene symbol	Gene type	Description	P-value^a^	ΔFPKM^b^
Up	LOC106513602	protein coding	heat shock protein 30-like	2.12E-28	4786
	pck1	protein coding	phosphoenolpyruvate carboxykinase 1	6.00E-28	2099
	LOC106513603	protein coding	heat shock protein 30-like	3.23E-22	1332
	LOC106520367	protein coding	heat shock cognate 71 kDa protein-like	1.06E-30	1028
	LOC106533043	protein coding	heat shock protein HSP 90-alpha-like	4.86E-22	926
	sqstm1	protein coding	sequestosome 1	9.59E-07	873
	LOC106523747	protein coding	apolipoprotein A-I-like	9.82E-20	697
	LOC106534642	pseudo	ubiquitin-like	3.83E-02	608
	LOC106535465	pseudo	ubiquitin-like	3.50E-06	585
	LOC106531886	protein coding	wiskott-Aldrich syndrome protein family member 2-like	1.43E-02	445
Down	ND6	protein coding	Mitochondrially encoded NADH:Ubiquinone Oxidoreductase Core Subunit 6	1.43E-04	-1595
	ND2	protein coding	Mitochondrially encoded NADH:Ubiquinone Oxidoreductase Core Subunit 2	6.28E-05	− 969
	ND1	protein coding	Mitochondrially encoded NADH:Ubiquinone Oxidoreductase Core Subunit 1	1.10E-02	− 823
	ND5	protein coding	Mitochondrially encoded NADH:Ubiquinone Oxidoreductase Core Subunit 5	2.47E-02	−775
	16S	rRNA	Mitochondrially encoded 16S ribosomal RNA	8.74E-03	− 441
	LOC106511533	protein coding	NADH-ubiquinone oxidoreductase chain 5-like	4.44E-02	− 429
	Pseudo16S	pseudo	Mitochondrially encoded 16S rRNA pseudogene	1.38E-19	− 219
	tRNALeu2–1	tRNA	Mitochondrial tRNA Leu2 copy 1	2.73E-41	−153
	tRNALeu2–2	tRNA	Mitochondrial tRNA Leu2 copy 2	1.10E-34	− 149
	txnip	protein coding	thioredoxin interacting protein	4.61E-08	−51

^a^FDR adjusted P-value

^b^Change in mean FPKM values of DII embryos in anoxia relative to DII t = 0

FPKM, Fragments per kilobase of transcript per million mapped reads

#### Gene expression following exit of DII

**Fig. 10:**
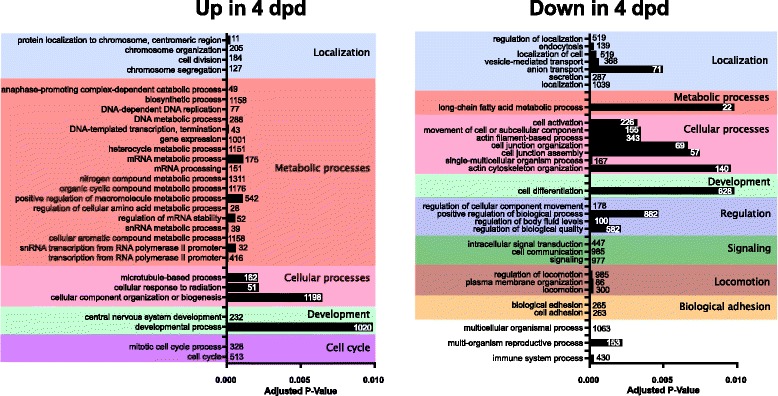
Significantly enriched GO terms based on differential abundance analysis of 4 dpd and normoxic DII embryos. Only terms with an adjusted P of < 0.01 are shown. Numbers in or near the bars indicate the total number of differentially expressed *A. limnaeus* genes associated with the respective GO term

Relative to normoxic DII embryos, we found 7600 genes to change in abundance in 4 dpd embryos (FDR, adjusted P < 0.05; Fig. 7; Additional file 1: Table S1G). In contrast to the comparison of transcript changes in anoxia, there is a roughly even split between number of genes that increase (4139) or decrease (3469) in abundance (Fig. 8a). There was very little overlap between the transcripts that are differentially expressed in anoxia and upon exit from diapause II (Fig. 8b). We observed several hemoglobin subunit transcripts to be highly increased in FPKM in 4 dpd embryos, while the top transcripts that decreased were generally more diverse (Table 9). GO enrichment analysis for the post-diapause II (4 dpd) embryo transcripts that were differentially expressed indicated enrichment for terms involved in localization, metabolic processes, cellular processes, and development (adjusted P < 0.01, Fig. 10; Additional file 1: Table S1H, I). Additionally, GO term enrichment for cell cycle was unique to transcripts that increased in abundance, while term enrichment for regulation, signaling, locomotion, and biological adhesion were unique for those that decreased in abundance.

**Table 9:** Top 10 genes in 4 dpd embryos with greatest transcript abundance changes relative to normoxic diapause II embryos

	Gene symbol	Gene type	Description	P-value^a^	ΔFPKM^b^
Up	LOC106528963	protein coding	hemoglobin embryonic subunit alpha	3.15E-38	4755
	LOC106528894	protein coding	hemoglobin subunit beta-like	4.10E-28	2717
	LOC106523751	ncRNA	uncharacterized	3.32E-26	2492
	LOC106512448	pseudo	fatty acid-binding protein, brain pseudogene	1.96E-08	2045
	LOC106525147	protein coding	hemoglobin subunit alpha-1-like	5.69E-45	1539
	LOC106522944	protein coding	fatty acid-binding protein, brain	1.31E-15	1483
	LOC106528904	protein coding	hemoglobin embryonic subunit alpha-like	1.01E-25	1370
	Pseudo16S	pseudo	Mitochondrially encoded 16S rRNA pseudogene	9.28E-05	1281
	LOC106525143	protein coding	hemoglobin subunit beta-like	8.06E-41	1079
	LOC106511047	protein coding	prothymosin alpha-B-like	9.16E-48	1062
Down	LOC106535866	protein coding	heat shock cognate 70 kDa protein	2.28E-16	−14,958
	LOC106517545	protein coding	lipocalin-like	1.35E-25	−10,327
	LOC106513764	ncRNA	uncharacterized	6.84E-06	−10,299
	ahsg	protein coding	alpha 2-HS glycoprotein	4.25E-11	− 6618
	LOC106524240	protein coding	galactose-specific lectin nattectin-like	1.47E-05	− 5070
	ccng1	protein coding	cyclin G1	6.56E-25	− 4354
	LOC106529138	protein coding	elongation factor 1-alpha-like	2.18E-04	− 4141
	LOC106518780	protein coding	keratin, type I cytoskeletal 19-like	1.06E-19	− 4062
	LOC106526789	protein coding	ferritin, middle subunit	3.16E-11	− 4003
	ubb	protein coding	ubiquitin B	8.06E-03	− 3830

^a^FDR adjusted P-value

^b^Change in mean FPKM values of DII embryos in anoxia relative to DII t = 0

FPKM, Fragments per kilobase of transcript per million mapped reads

#### Transcriptional expression of stress response genes

**Fig. 11:**
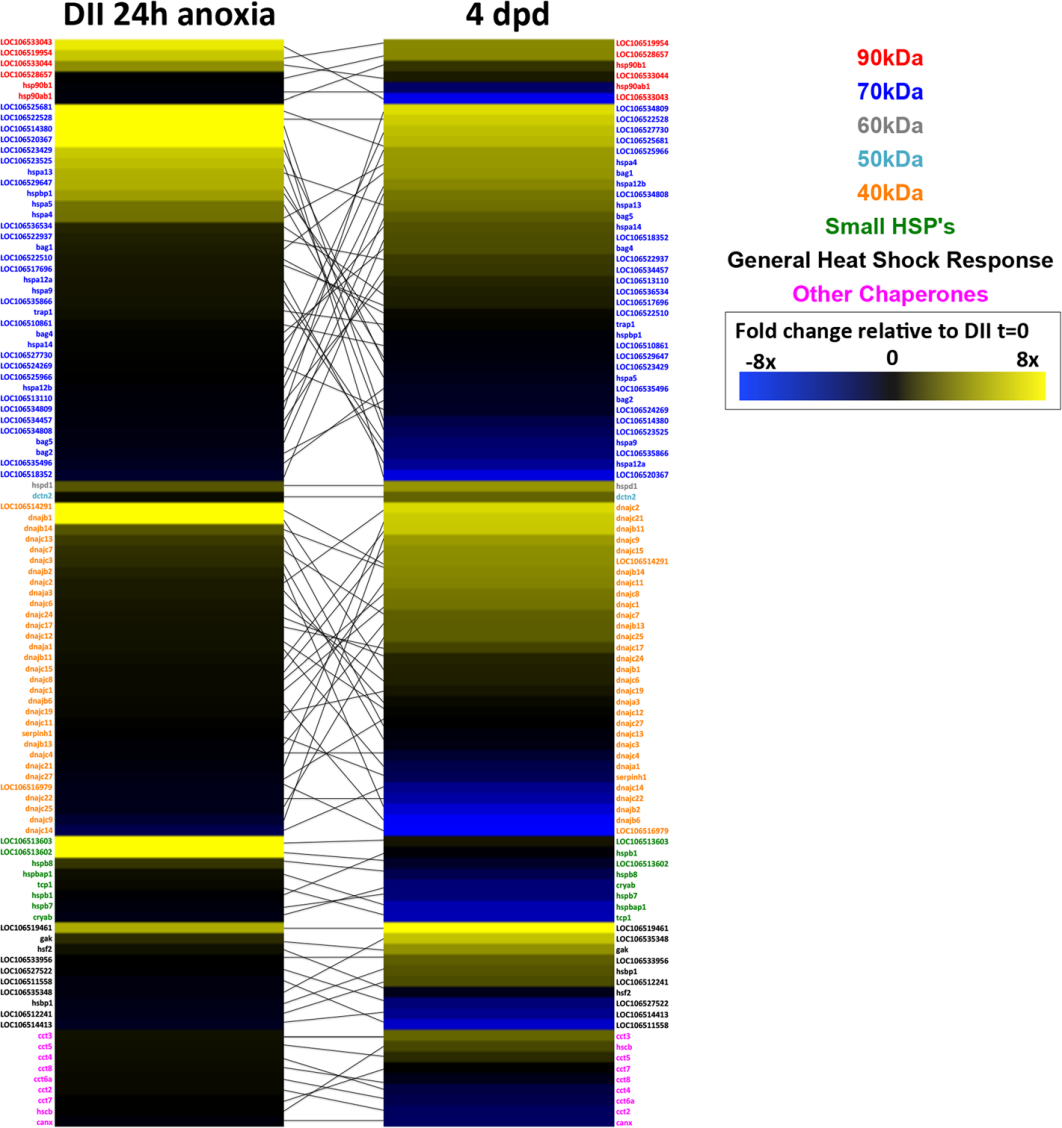
Abundance of heat shock protein related transcripts in anoxic DII embryos and 4 dpd embryos. Genes shown are relative to normoxic DII embryos and have significant differential abundance (FDR adjusted p-value < 0.05). Genes within families were ordered by highest to lowest fold-change for each comparison. Lines between the two heat maps connect the same genes. Fold changes for the heat map are log_2_ transformed

**Fig. 12:**
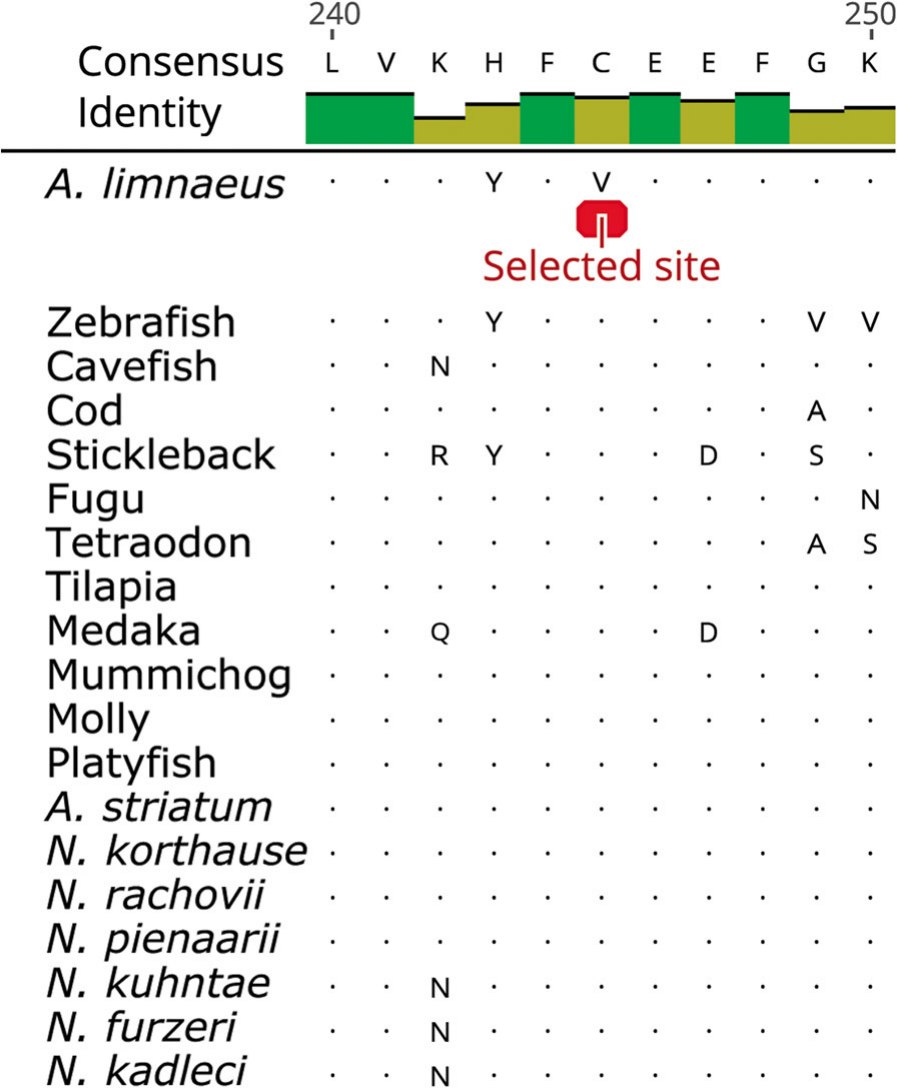
Representative multiple species alignment of hspa4 showing the selected site in *A. limnaeus.* Genes were aligned using PRANK and filtered using GUIDANCE. The alignment was visualized using Geneious software. Residue locations are relative to the start of the filtered alignment consensus

When compared to normoxic DII controls, we observed 101 genes related to heat shock proteins (hsps), accessory heat shock proteins, or related to the general stress response to be significantly differentially expressed in both anoxia and post-DII development (Fig. 11). Although there were not any apparent trends in expression profiles between the two comparisons, a majority of the significantly differentially expressed 70-kDa heat shock protein (hsp70) family transcripts had an inverse expression profile between these comparisons (Additional file 1: Table S1J). The gene hspa4, also a part of the hsp70 family, was also determined to be under positive selection by PAML analysis. In *A. limnaeus*, the residue change in hspa4 appears to be from an ancestral cysteine to a valine, amino acids that are polar and nonpolar, respectively (Fig. 12).

## Discussion

### Size, structure, and annotation quality of the *A. limnaeus* genome

The *A. limnaeus* nuclear genome has been previously reported to contain 22 haploid chromosomes [38]. However, direct or sequence-based measurements of genome size in this species has not yet been determined. The *A. limnaeus* genome is estimated to be slightly larger than the cod genome, but much smaller than *N. furzeri* and *D. rerio*. We observed the *A. limnaeus* genome to be highly repetitive, and our assembly benefitted heavily from the use of mate-pair libraries for scaffolding. Interestingly, nearly half of the repetitive elements in the *A. limnaeus* genome were unable to be classified, a result similar to findings in *Nothobranchius* and another South American annual killifish, *Austrolebias charrua* [39, 40]. Comparing these unclassified repeat elements between annual killifish species may reveal possible conserved mechanisms of genome expansion and evolution in killifishes. Despite the highly repetitive structure of the *A. limnaeus* genome, BUSCO analysis examining 3023 conserved vertebrate genes indicates that the *A. limnaeus* draft genome has completeness and fragmentation similar to the genomes of *N. furzeri* and *K. marmoratus*, two closely related species assembled with similar strategies. Overall, the *A. limnaeus* genome currently contains highly complete gene models, but may benefit from longer reads in the future to patch the extensively repetitive regions occurring between genic sites.

### Several mitochondrial related genes may be under positive selection in *A. limnaeus*

Our phylogenetic tree places *A. limnaeus* sister to the Family Nothobranchiidae, and nested within the Order Cyprinodontiformes, which agrees with previous reports [32, 34]. PAML analysis identified 105 genes to have at least one significantly selected site along the *A. limnaeus* branch. Several GO terms of relevance to annual killifish biology are significantly enriched among this list of genes, including stress tolerance and oxidation-reduction activity [21, 41]. Reichwald et al. [31] found seven genes under positive selection along the *N. furzeri* branch and one along the *N. pienaari* branch, but we did not find the same orthologs for these *Nothobranchius* species to also be under selection in the *A. limnaeus* branch using our analysis. When compared to the list of genes under putative positive selection in *N. furzeri* by Valenzano et al. [30]*,* the eight shared positively selected genes had selected sites both inside and outside of predicted protein domain regions. This suggests that selection may be operating at the level of domain function as well as the tertiary structure of the proteins, and while some genes may be positively selected in both lineages, there may also be extensive differences in which genes are under selection in other annual killifish lineages.

In a recent report by Sahm et al. [42], multiple nuclear genes involved with mitochondrial biogenesis or activity were also found to be under putative positive selection along several *Nothobranchius* branches associated with shortened lifespan. Our results suggest that only NADH:Ubiquinone Oxidoreductase Subunit S5 (ndufs5) is under putative positive selection along both the *A. limnaeus* branch and the *Nothobranchius* branch containing the last common ancestor of *N. pienaari* and *N. rachovii* previously reported by Sahm et al. [42]. However, the annual killifish phenotype is a complex mix of several phenotypes and we note that our strain of *A. limnaeus* does not exhibit the shortened lifespan of several *Nothobranchius* species, nor do *Nothobranchius* species appear to have the extreme anoxia tolerance of *A. limnaeus* (Polačik and Podrabsky, unpublished). These results raise the possibility that the proteins under positive selection in *A. limnaeus* or in *Nothobranchius* species may be involved in different aspects of annual killifish biology, such as with stress tolerance in *A. limnaeus*, and aging in several *Nothobranchius* species.

Interestingly, four of the genes under putative positive selection include NADH:Ubiquinone Oxidoreductase accessory subunits that are a part of complex I, an enzyme complex located in the inner mitochondrial membrane that is also the first enzyme of the mitochondrial electron transport chain [43]. Recent evidence has suggested that several complex I subunits encoded by the *A. limnaeus* mitochondrial genome (mtgenome) have apparent insertions or deletions at the N-terminus when compared to multiple other teleost species within the Order Cyprinodontiformes [44]. One of these mtgenome-encoded complex I subunits, ND5 contains a unique 11 amino acid insertion in the N-terminus and is thought to interact directly with one of the complex I accessory subunits we have identified to be under putative positive selection, ndufb6 [45]. Importantly, complex I is thought to be a primary source of reactive oxygen species (ROS) generation, and control of ROS production is likely essential for surviving anoxia [46–48]. Because mitochondria perform essential energy-generating processes, it is likely that nuclear genome evolution will favor mutations that can accommodate changes in mtgenome sequence [49]. However, it is unclear if this is a case of mitonuclear co-evolution to promote the unique stress-tolerant phenotype of *A. limnaeus*, or compensation of nuclear genes to prevent complex I dysfunction. Further investigations into the role of the complex I subunits and possible roles in ROS production or suppression may yield important insight into the evolution of dormancy and anoxia tolerance in this lineage.

### DII embryos have a transcriptional response to anoxia treatment

We were interested in gene expression changes that may facilitate extreme anoxia tolerance in *A. limnaeus* DII embryos, Therefore, we used RNAseq to compare transcript abundance in DII embryos after 24 h of anoxia relative to normoxic DII embryos. Following anoxia treatment, we observed that the majority of transcripts do not have a significant change in abundance. This observation suggests an overall stabilization of the transcriptome during exposure to anoxia consistent with available data and theory [50–52]. Unexpectedly, 294 transcripts increased in abundance, supporting active regulation of a small portion of the transcriptome through either increased synthesis or possibly differential stabilization. Not surprisingly, many of the genes with the largest increases in abundance are associated with the cellular stress response. Of note, two hsp30-like transcripts ranked as numbers 1 and 3 in transcripts that increased in abundance. Others have reported on the importance of hsp30 proteins in the heat shock response of desert fishes, although their functional importance has not been experimentally explored [53, 54]. Relatively fewer transcripts decreased in abundance, and many of these genes are indicated by GO term enrichment analysis to be associated with metabolism. This suggests a trend towards further decreasing metabolic processes during anoxia even though the embryos are already in a highly metabolically depressed state.

Interestingly, the majority of transcripts in anoxic DII embryos that had the greatest decreases in FPKM relative to normoxic controls are associated with the mitochondrial transcriptome. Previous work has suggested that regulation of mtgenome-encoded ND5 through differential stabilization of *ND5* mRNA, rather than mitochondrial DNA copy number, may be the rate-limiting step in the control of cellular respiration [44, 55–58]. While we did observe a significant depression of *ND5* transcripts in anoxic DII embryos, we also found significantly lower abundance of *ND1*, *ND2*, and *ND6* transcripts as well, which indicates that reduced respiration in *A. limnaeus* may be controlled through a coordinated reduction of multiple complex I subunit transcripts rather than just *ND5*. Transcript levels of 16S rRNA decrease during anoxia in *A. limnaeus*, which could lead to a reduction in capacity for mitochondrial protein synthesis. In contrast, transcript abundance for several mitochondrial genes increases in the anoxic turtle brain, suggesting that their mitochondria actually respond by increasing transcription [50]. This divergence in the transcriptome implies different mitochondrial responses to long-term anoxia between turtles and *A. limnaeus* embryos. We also found seven nuclear-encoded proteins with residues under putative positive selection that are involved with mitochondrial translation. However, it is unclear if these proteins have novel functional significance, or if they represent nuclear compensation for rapidly evolving mitochondrial rRNAs following the unique and potentially isolating population dynamics of *A. limnaeus* [59].

### Transcriptional response to diapause termination

While previous work has examined transcriptional changes that may be essential for entrance into DII in annual killifish [31], it is currently unclear how DII embryos may reactivate metabolism and development through changes in RNA expression. The transcript we observed with the greatest decrease in abundance in post-diapause II embryos is a heat shock cognate 70 (*hsc70*) protein with a mean change of − 14,958 FPKM. High abundance of this transcript in diapausing embryos could initially be interpreted to play a role in the increased tolerance to environmental stress observed in these embryos. However, DII and 4 dpd embryos have a similar tolerance of anoxia, with 4 dpd embryos possibly having even higher tolerance, which would suggest a different role for this transcript if the high abundance is translated to high levels of proteins [11]. Interestingly, previous work has shown that hsc70 overexpression promotes degradation of ion channels as they are trafficked through the cell [60]. DII embryos are characterized by an exceptionally low ion permeability that decreases steadily during post-DII development [10], and therefore reduced hsc70 expression may promote increased routing of ion channels to cell membrane surfaces. This would be a unique function for hsc70 in fish embryos that has not yet been described or explored.

Another gene of interest that decreases significantly during post-diapause II development is cyclin G1. This cyclin is known to interact with p53 and is associated with cell cycle arrest at a number of different cell cycle checkpoints [61, 62]. Further exploration of the functional significance of this transcript and its signaling networks may lead to the key players that regulate exit from the cell cycle during diapause in this species.

Importantly, most of the transcripts that change drastically during PDII development very likely have little to do with the regulation of diapause, but rather are due to the resumption of active development and the associated changes in tissue differentiation and morphogenesis. For example, several hemoglobin transcripts are among the top ten genes with the greatest increase in abundance compared to diapause II embryos. This increase in abundance coincides with the appearance of red blood cells in the embryos and likely supports their transition to aerobic metabolism as they continue development [7, 22]. A more thorough and mechanistic analysis of pre- and post-diapause expression patterns will be required to identify putative regulators of metabolic dormancy.

### Heat shock proteins in annual killifish anoxia tolerance, development, and evolution

Our two RNA-seq comparisons provided a unique opportunity to study genes that may have roles in either stress tolerance or embryonic development, and in some cases, both. Between these two RNA-seq analysis comparisons (DII 24 h anoxia or 4 dpd embryos relative to DII normoxia), we found 101 genes related to hsps, accessory heat shock proteins, or related to the general stress response, to be differentially expressed in both comparisons. Hsps are a conserved group of molecular chaperones that function to facilitate proper folding of newly translated or misfolded proteins during normal and stressed states, but also can have several other functions related to cell cycle, homeostasis, signaling, and apoptosis [63–65]. We observed differential expression of several hsps from the 90, 70, 60, 50, 40, and small kDa classes in both comparisons, and in the anoxia-treated DII embryos we also found a significant GO term enrichment for genes involved in protein refolding and response to topologically incorrect proteins. Heat shock proteins from the 30 kDa, 70 kDa, and 90 kDa families were represented in the list of top 10 transcripts with the greatest increase in abundance in response to anoxia in diapausing embryos and are likely a direct response to stress. However, we also observed a diversity of hsp transcripts within the 70 kDa and 40 kDa families that increased in abundance during post-diapause II development. Notably, 20 out of 35 hsp70 family transcripts had an inverse abundance profile when the two groups were compared, suggesting stage-specific roles in development that may be independent of stress tolerance. Thus, *A. limnaeus* may be a powerful model for teasing apart the role of hsps in normal development and stress tolerance, both of which likely contribute in major ways to buffering and canalization during vertebrate development.

One of the molecular chaperones that increased in expression for both comparisons is also under putative positive selection in *A. limnaeus* and *N. furzeri* [30]. This gene, hspa4, is a molecular chaperone from the well-studied hsp70 family, and although it was initially thought to be interchangeable with hsc70, it has been previously shown to have separate cellular functions [60]. In hsp70 family proteins, key replacements of cysteine residues with other amino acids has been reported to reduce redox sensitivity and alter responses to oxidative stress (Miyata et al. 2012). Interestingly, the presence of an inducible hsp70 isoform that is constitutively expressed during *A. limnaeus* early embryonic development and diapause has been previously reported [66]. We found that *hspa4* transcript abundance increased by 26 FPKM (~ 2.5-fold) in anoxic DII embryos. An increase in hsp70 following anoxia has also been reported in the brains of freshwater turtles, but not the brine shrimp *Artemia franciscana,* and thus may be a conserved response in vertebrates [57, 67]. We also found a significant increase (37 FPKM, ~ 3-fold) in abundance of *hspa4* transcripts in post-DII (4 dpd) relative to normoxic DII embryos. Importantly, expression of inducible hsp70 is usually not present in teleost embryos during non-stressful conditions [68–70]. It is possible that unstressed 4 dpd embryos increase expression of *hspa4* during normal development to provide a rapid response to environmental insults, thus suggesting a unique survival strategy among developing aquatic embryos. Taken together, these data indicate that *hspa4* has both physiological and evolutionary importance for stress tolerance, development, and evolution in *A. limnaeus* biology and is an interesting gene for future studies.

## Conclusions

Our release of the *A. limnaeus* genome is the first publicly available South American annual killifish genome and complements recent genome publications of the African annual killifish *Nothobranchius furzeri* by two independent research groups [30, 31]. Despite the high repeat content of the *A. limnaeus* genome, the combination of a merged de novo assembly approach and a vast amount of RNA-seq data has allowed us to generate a draft *A. limnaeus* genome with high-confidence gene annotations, on par with other teleost assemblies. This genome assembly is a critical tool for exploration of annual killifishes at the molecular and genetic levels, and will likely significantly accelerate advances in our understanding of this unique group of vertebrates.

## Methods

### Genome size estimation by flow cytometry

Zebrafish and chicken blood were generously provided by Dr. Kim Brown (Portland State University) and Chrissie Zaerpoor (Kookoolan Farms, Yamhill, OR), respectively. Whole blood from zebrafish (*n* = 2), chicken (*n* = 1), or *A. limnaeus* (*n* = 3) was homogenized in 1 ml of Galbraith buffer (30 mM sodium citrate, 45 mM MgCl_2_, 20 mM MOPS, 0.1% Triton-X 100, 1 mg ml^− 1^ RNAse A, pH 7.2) on ice using a Sorvall Omni-mixer (speed setting 2) and a loose-fitting Teflon pestle for about 20 strokes. Three technical replicates of the chicken blood were analyzed. Homogenates were filtered through a 35 μm mesh screen by centrifugation at 100 x *g* for 1 min at 4 °C. Dissociated nuclei were stained in 1 mg ml^− 1^ propidium iodide for 30 min at room temperature and fluorescence intensity were measured using the BD Accuri C6 flow cytometer at a flow rate of 14 μl min^− 1^ for 100,000 counts (BD Biosciences, San Jose, CA). Counts were gated manually to remove noise from cellular debris. Fluorescence peaks were identified and analyzed with the included BD CSampler Software. The genome size of *A. limnaeus* was inferred from linear regression of zebrafish and chicken nuclei fluorescence relative to assembled genome lengths for those two species, including all gaps (Ensembl release 77).

### Total genomic DNA extraction of adult tissue

#### DNA extraction of adult tissues

Adult *A. limnaeus* fish used for sequencing and for spawning were cared for as previously described by Podrabsky (1999) and in accordance with an approved Portland State University Institutional Animal Care and Use Committee protocol (PSU protocol #33). The tissue used for single-individual Illumina sequencing was derived from an adult *A. limnaeus* male (3 months post-hatch, 3.7 g wet mass). The fish was euthanized by immersion in an ice bath for approximately five minutes until fully unresponsive to stimuli followed by cervical transection. Liver, white muscle (skin and scales removed), and brain tissue were removed and transferred to DNA extraction buffer (10 mM Tris, 100 mM EDTA, 0.5% SDS, 200 μg ml^− 1^ Proteinase K (Thermo Scientific #EO0491), pH 8.0) at a ratio of 1 mg tissue per 10 μl buffer. Tissues were gently homogenized using a Teflon pestle in 1.5 ml microcentrifuge tubes and incubated for 3 h at 50 °C with agitation every 20–30 min. DNA was extracted by briefly vortexing the homogenates with 1 vol phenol (equilibrated with 10 mM Tris-HCl pH 8.0, 1 mM EDTA) followed by centrifugation at 5000 x *g* for 10 min at 4 °C. The aqueous phases were collected using a wide-bore pipette and extracted with phenol again as described above. Following the second phenol extraction, the aqueous phase containing the DNA was extracted a third time by gentle mixing with 1 volume of chloroform:phenol (1:1) followed by centrifugation at 5000 x *g* for 10 min at 4 °C. The aqueous phase was collected and the DNA precipitated by the addition of NaCl (final concentration of 200 μM) and 2 volumes of 100% EtOH followed by gentle mixing via tube inversion. DNA was pelleted by centrifugation at 16,000 x *g* for 10 min at 4 °C. The DNA pellet was washed twice with 1 ml 70% EtOH. After each wash, the DNA was pelleted by centrifugation at 16,000 x *g* for 5 min at 4 °C. The air-dried (5–10 min at room temperature) DNA pellet was resuspended in 500 μl of RNAse buffer (10 mM Tris, 5 mM EDTA) with 100 μg ml^− 1^ DNAse-free RNAse A (Thermo Scientific #EN0531) and incubated at 37 °C for 45 min with occasional tube inversion. The samples were extracted twice more as described above with chloroform:phenol (1:1) followed by precipitation using 0.1 vol of 3 M sodium acetate (pH 5.2), and 2 vol of 100% EtOH. DNA was pelleted by centrifugation at 16,000 x *g* for 10 min at 4 °C and the pellet was washed twice with 1 ml 70% EtOH as described above. The DNA pellets were dried briefly at room temperature and resuspended in 100 μl of buffer EB (10 mM Tris-Cl pH 8.5, Qiagen #19086) at 37 °C with occasional agitation until pellets dissolved completely.

The tissue for mixed-individual Illumina sequencing was collected from three adult male fish. Fish were euthanized as described above and whole brains were extracted. The three brain tissues were extracted using the Qiagen DNeasy Blood and Tissue Kit (Qiagen #69504) according to the manufacturer’s instructions.

#### DNA quantification and quality assessment

DNA purity was determined by observation of A_260_/A_280_ ratios between 1.8–1.9 using an Infinite M200 Pro plate reader equipped with a NanoQuant plate (Tecan, San Jose, CA, USA), 2 μl of sample, and default software settings (i-control software, Tecan). DNA concentration was determined by using the Quant-iT dsDNA Assay Kit, broad range (Thermo Fisher Scientific #Q33130) according to manufacturer’s instructions. DNA integrity was confirmed by observation of high-molecular weight DNA above 20 kb following electrophoresis of 1 μg of total DNA on a 1% agarose gel.

### Sequencing and read quality control

#### Illumina sequencing

DNA sequencing libraries were prepared and sequenced at the University of Oregon High Throughput DNA sequencing and Genomics facility. Purified DNA was sonicated to an average size of 170 bp and was prepared for sequencing using the Nextera library prep kit (Illumina). Larger fragments were sonicated into size ranges of 2 kb – 3 kb, 5 kb – 8 kb, and 10 kb – 15 kb. These large fragments were size-selected by gel electrophoresis and were used for mate-pair libraries (Additional file 2: Table S2A). Fragment and mate-pair libraries from the single *A. limnaeus* male were sequenced on the Illumina Hi-Seq 2500 platform with 101 bp paired-end reads. Mate-pair libraries from the mixed individuals were sequenced on the Illumina Hi-Seq 2000 platform with 101 bp paired-end reads.

#### Read quality control

Adapters were removed from Illumina reads using Trimmomatic v0.33 [71] in palindrome mode and using the included Illumina adapter list (seed mismatches = 2, palindrome clip threshold = 30, simple clip threshold = 7, minimum adapter length = 1, keep both reads = true). Error-correction was performed using the Allpaths error correction module [36]. For scaffolding, mate-pair libraries were further trimmed to 37 bp in length to reduce instances of chimeric reads and remaining reads with quality scores >Q10 across the entire read were used.

### Genome assembly strategy

**Fig. 13:**
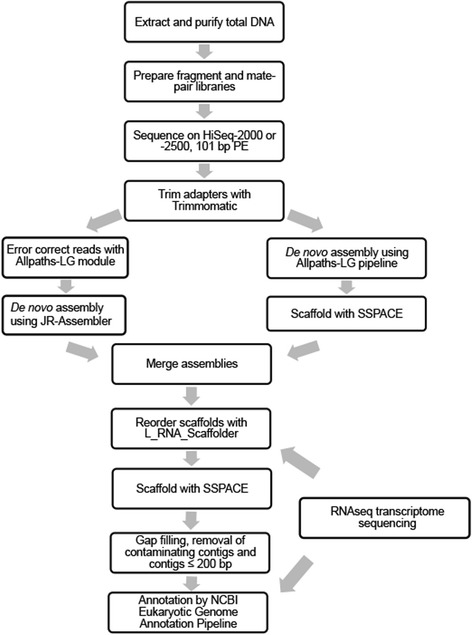
Flowchart for assembly and annotation of the *A. limnaeus* genome. The *A. limnaeus* genome was assembled by merging assemblies from Allpaths-LG and JR-assembler. Scaffolds were formed using SSPACE and L_RNA_Scaffolder. The draft genome was then annotated using the NCBI Eukaryotic Genome Annotation pipeline

A summary of the assembly and annotation strategy used for the *A. limnaeus* genome is shown in Fig. 13. Adapter-free reads were de novo assembled using two algorithm strategies. The library types and insert sizes used for genome assembly are shown in Additional file 2: Table S2A. First, the untrimmed reads were assembled using the Allpaths-LG pipeline version r44837, which is based on a modified de Brujin graphing algorithm [36]. A total of 707,851,166 fragment library and 678,076,430 jumping library adapter-free reads were used as input into Allpaths-LG. As suggested by the Allpaths-LG manual, reads that failed to pass the Illumina purity filter were retained in the read pool for de novo assembly. In a separate assembly, the fragment reads were error corrected using Allpaths-LG and de novo assembled using JR-assembler, an assembler that is based on read overlap to extend contigs [72]. Prior to assembly with JR-assembler, reads were trimmed to 90 bp and filtered using the included mdust algorithm. Contigs were ordered into scaffolds within the individual assemblies by using SSPACE Basic 3.0 using the mate-pair libraries listed in Additional file 2: Table S2A and settings that required at least three connections with 70% identity to scaffold two contigs (−k = 3, −a = 0.7) [73]. The resulting scaffolds were then merged between Allpaths-LG and JR-assembler to provide the best representation of unique scaffolds from both assemblies. L_RNA_Scaffolder software was used to improve the assembly by breaking apart and rejoining poorly supported scaffolds using a preliminary transcriptome derived from a 12 dpd embryo RNA-seq dataset (Riggs and Podrabsky, unpublished) [74]. The assembly was run through a final round of SSPACE Basic v3.0 and a custom script based on IMAGE (Tsai et al. 2010) was used to close gaps. The *Austrofundulus limnaeus* genome v1.0 has been screened and cleaned of contaminating contigs, and all contigs 200 bp or smaller were removed.

### Automated annotation by NCBI

The NCBI eukaryotic annotation pipeline integrates ab initio gene modeling and protein/transcript/RNA-seq alignments to annotate genomes. The *A. limnaeus* genome v1.0 was submitted to NCBI and annotated using version 6.4 of this pipeline. A total of 114 RNA-seq biosamples (9,297,509,096 RNA-seq reads) from various treatments and stages of *A. limnaeus* embryonic development were obtained via SRA and used for gene prediction (Additional file 2: Table S2B).

Libraries of trimmed sequence reads (see methods below) grouped by stages and treatment were assembled de novo into transcriptome assemblies using Trinity software (version 2.0.6) [75], using a default k-mer length of 25 nucleotides (nts) for overlapping into contiguous sequences and the minimum contiguous sequence length setting (min_contig_length) at 200 nts. Assembled sequences have been deposited into the NCBI Transcriptome Shotgun Assembly (TSA) database with the TSA records listed in Additional file 2: Table S2C.

### Analysis of genome assembly

Genome completeness of *Austrofundulus limnaeus* v1.0 was estimated with CEGMA version 2.5 on the CEGMA virtual machine v1.0 [76] http://korflab.ucdavis.edu/datasets/cegma/cegma_vm.html). CEGMA scans genomic sequences for homologous sequences to 248 highly conserved eukaryotic genes. Genome completeness was also estimated with BUSCO version 1.22 using the vertebrata BUSCO profile [77]. BUSCO dependencies included AUGUSTUS v2.2.2, BLAST+ 2.2.28, and HMMER v3.1b2. Repetitive DNA elements in the *A. limnaeus* genome, such as transposons, were identified with RepeatModeler 1.0.8 [78].

### Protein clustering analysis

Protein coding sequences (CDS) were downloaded from BioMart (Ensembl Genes release v82) or Genbank. CDS inferred from transcriptome assemblies were also used for several *Nothobranchius* species and one non-annual killifish species, *Aphyosemion striatum* (Additional file 1: Table S1A). CDS were translated to amino acid sequences and only the longest isoform from each gene was retained for clustering analysis. Gene family clusters were inferred using Proteinortho v5.11 with default settings [79].

### Phylogenetics

Genes identified as single-copy orthologs that were included in high-confidence clusters (Proteinortho Alg.-Conn score = 1) were aligned using MAFFT v7.158b and trimmed with GBLOCKS [80, 81]. The best-fit amino acid substitution model was determined using the resulting alignment as input to ProtTest 3.4.1 [82]. Phylogenetic inference using maximum-likelihood on this alignment was performed using PhyML v3.1 with the JTT + I + G protein substitution model and 100 bootstrap replications [83]. Based on findings by the Ensembl Compara team, our resulting tree was rooted by setting Zebrafish and Cavefish as outgroups (http://dec2016.archive.ensembl.org/info/about/speciestree.html).

### Positive selection analysis

For positive selection analysis, we considered all gene clusters identified by Proteinortho that included an ortholog from *A. limnaeus*, *N. furzeri*, the non-annual killifish *A. striatum,* at least one other *Nothobranchius* species, and two other teleost fish (a minimum of six single copy genes from all the species). Coding sequences in the filtered clusters were aligned using PRANK v.140603 [84]. Poorly aligned regions were trimmed using GUIDANCE v2.0 [85]. Genes and individual amino acids under positive selection in the *A. limnaeus* lineage were then identified using the branch-site model in CODEML implemented in the Phylogenetic Analysis by Maximum Likelihood package (PAML) [86]. The phylogenetic tree generated using PhyML was used as the input guide tree (Fig. 2. To generate a high confidence list of genes and sites under possible positive selection in *A. limnaeus*, we considered genes that were (1) identified by PAML to have at least one site under selection along the *A. limnaeus* branch, (2) did not have gaps within ±5 amino-acids from the putatively selected sites, and (3) had an FDR-corrected *p*-value of less than 0.2 (< 20% FDR) for the sites. In our analysis, an FDR of 0.2 corresponded approximately to an uncorrected *P*-value of 0.01. We compared the resulting list of positively selected proteins in *A. limnaeus* to ones previously reported to be under positive selection in *N. furzeri* using an independently annotated genome [30]. To validate orthology between the two *N. furzeri* annotations and *A. limnaeus*, we performed an additional Proteinortho analysis that omitted the annual killifish transcriptomes but included both *N. furzeri* proteomes. This second Proteinortho output was then used to ensure that the proteins reported to be under positive selection in the *N. furzeri* genome by Valenzano et al. [30] were in valid clusters that also contained expected orthologs of the NCBI annotated *N. furzeri* genome as well as *A. limnaeus*. Finally, *A. limnaeus* proteins that were also under putative positive selection in *N. furzeri* were used as input into InterProScan 5.24–63.0 (https://www.ebi.ac.uk/interpro/interproscan.html) to predict protein domains.

### Embryo sampling and poly-A RNA sequencing

*A. limnaeus* embryos were collected from spawning adults according to previously established husbandry methods [35]. Embryos were maintained at 25 °C in darkness until DII [15, 17, 22]. For anoxia exposures, DII embryos were exposed to 24 h of anoxia at 25 °C in a Bactron III anaerobic chamber (Sheldon Manufacturing, Cornelius, OR) [87]. To obtain post-diapause II embryos, diapause was experimentally broken by exposing embryos to continuous light for 48 h at 30 °C. Embryos were then returned to 25 °C in darkness until staging at 4 days post diapause (dpd) [22]. For each treatment (DII normoxia, DII 24 h anoxia, and 4 dpd) four biological replicates (*n* = 4), comprised of 20 embryos each, were flash-frozen and stored at − 80 °C until RNA extraction. Total RNA was extracted using TRIzol reagent (Invitrogen Inc., Carlsbad, California) as previously described [88]. cDNA libraries were prepared using the Illumina TruSeq RNA Sample Preparation kit (v2, Illumina, San Diego, CA, USA) following the manufacturer’s instructions with 1 μg of total RNA as starting material. The purified cDNA libraries were quantified by qPCR and their quality was confirmed by a 2100 Bioanalyzer (Agilent Technologies, Santa Clara, CA, USA) using a DNA 1000 chip. The libraries were sequenced (100 nt paired-end reads, 4 samples multiplexed per lane on the flow cell) on an Illumina HiSeq 2000 at Oregon Health & Science University.

### Analysis of poly-a RNA sequence data

The following analyses were performed in a UNIX environment on the Portland State University computing cluster. Sequence quality was initially assessed using FastQC, version 0.10.1 [89] to ensure high quality data. Sequence reads were filtered on quality scores and trimmed for the presence of adapter sequences using Trimmomatic [71] with the settings “ILLUMINACLIP:2:30:7:1:true”, “SLIDINGWINDOW:5:15”, “LEADING:20”, “TRAILING:20”, and “MINLEN: 25”. Quality reads were mapped to the *A. limnaeus* genome 1.0 using the *--very-fast-local* preset in Bowtie2 [90]. Preserved paired reads after trimming were aligned in paired-end mode and any orphaned mates after trimming were aligned in single-end mode. These data sets were deposited into the SRA with the accession numbers listed in Additional file 2: Table S2D. Reads that aligned to the *A. limnaeus* nuclear and mitochondrial [44] genome with 0 mismatches were used for expression analyses. Transcript counts per gene (union mode) were generated for all samples using the *summarizeOverlaps* function of the GenomicAlignments package from Bioconductor [91] and the NCBI *A. limnaeus* Genome Annotation Release 100. Count matrices were filtered for genes with 1 or more normalized counts summed across all replicates. Gene abundances were calculated as FPKM and differential expression analysis was performed using DESeq2 in the R Bioconductor package. Differential gene expression was determined on gene count data using the negative binomial distribution and estimations of mean-variance dependence [92] using a Benjamini-Hochberg multiple comparisons adjusted FDR of 5%.

### Gene ontology enrichment analysis

For differentially expressed genes and genes identified as being under possible positive selection in *A. limnaeus*, we inferred gene ontology (GO) terms using human orthologs. A BLASTp search against *A. limnaeus* proteins was run using human proteins obtained from Ensembl (Assembly GRCh38, Release 37). The top hit for each gene with a minimum e-value of 10^− 5^ was used to infer orthology. GO terms for the orthologous genes were obtained using the online UniProt Retrieve/ID mapping tool. We used hypergeometric test implemented in the Bioconductor package GoStats [93] to determine GO term enrichment. For the genes under possible positive selection, we used all the filtered genes input into PAML as background. For RNA-seq, genes with an average FPKM of at least 2 in either of the groups being compared were used as background.

## Additional files

Additional file 1: Spreadsheet containing **Tables S1A-J.** The spreadsheet contains supplemental information on the species used for clustering analysis, positive selection results, GO term enrichment results, differential transcript abundance results, and Hsp70 family transcript abundance results. (XLSX 997 kb)

Additional file 2: Spreadsheet containing **Tables S2A-D.** The spreadsheet contains information on the libraries used for assembly and annotation of the *A. limnaeus* genome. In addition, the spreadsheet contains information on the RNA-seq libraries used for differential expression analysis. (XLSX 65 kb)

## Abbreviations

DI: diapause I
DII: diapause II
DIII: diapause III
dpd: days post-diapause II
FDR: false discovery rate
FPKM: Fragments Per Kilobase of transcript per Million mapped reads
GO: gene ontology
hsps: heat shock proteins
mtgenome: mitochondrial genome
PDII: post-diapause II
ROS: reactive oxygen species
